# Extravascular Temperature Control for Heatstroke With Multi‐Organ Failure: A Survivor's Case Report

**DOI:** 10.1002/ccr3.70131

**Published:** 2025-01-29

**Authors:** Van Ha Thi Bich, Lich Nguyen Duc, Anh Tran Ngoc

**Affiliations:** ^1^ Phu Tho Provincial General Hospital Phu Tho Vietnam

**Keywords:** critical care, extravascular temperature control, heatstroke, multi‐organ failure, plasma exchange

## Abstract

Heatstroke is caused by a loss of control over body temperature. There is a high risk of death if it is not treated quickly and properly. In this article, we report a clinical case of a 21‐year‐old male patient treated for heatstroke with extravascular temperature control. The patient lost consciousness after being out in the sun for more than 2 h. His body temperature was 41°C upon admission to the district hospital, and then 4 h later, he was transferred to our department. The patient was administered extravascular temperature control with a target temperature of 33°C within 24 h. We rewarmed at a rate of 0.25°C/h to 37°C within 16 h, and the patient was taken off sedation to assess consciousness. After 2 days, the patient's consciousness improved from GCS 5 to 10, and the patient maintained a body temperature of 37°C for another 4 days. However, the patient still had liver failure and severe coagulation disorders on day 5. He was given fresh frozen plasma or plasma exchange. The patient was extubated after 8 days and discharged from the hospital after 24 days of treatment. In conclusion, extravascular temperature control can be used effectively to treat heatstroke, combined with the treatment of damaged organs.

**Summary**

- Extravascular temperature control can be used effectively for heatstroke with multi‐organ failure, along with supportive therapy.

## 1 Introduction

Heatstroke is diagnosed clinically by excluding other causes. This is a life‐threatening condition that requires prompt, correct treatment, and has a high mortality rate [1]. Heatstroke is accompanied by increased body temperature (> 40°C) and loss of consciousness or coma [2]. It mainly occurs when excessive heat is absorbed from outside the body, occurring in cases of working or staying in hot, humid environments for long periods of time or when exceeding the body's ability to remove heat [3].

Temperature control aims to improve neurological outcomes in heatstroke patients by reducing brain metabolism, inhibiting harmful inflammatory reactions, and reducing brain edema, which was previously commonly known in the treatment of patients in a coma after circulatory arrest [4]. In this article, we report a case of heatstroke treated with extravascular temperature control.

## 2 Case History

**FIGURE 1:**
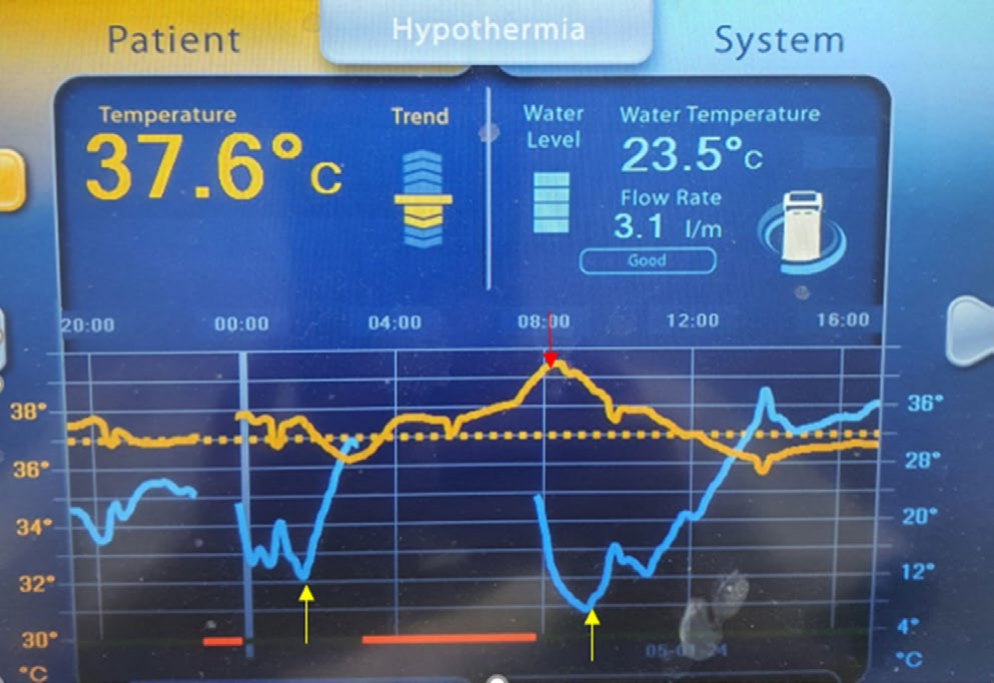
Temperature of the patient during hypothermia, water temperature is indicated by the yellow arrow, and patient temperature is indicated by the red arrow.

A 21‐year‐old previously healthy male patient was admitted to the emergency department of the district medical center due to unconsciousness after riding a motorbike in the sun for 2 h and transferred to our department of intensive care after 4 h (Figure 1). The patient was on his way home from Hanoi, Vietnam, to Phu Tho, Vietnam, sitting on the back of a friend's motorcycle between 1:00 p.m. and 3:00 p.m. on April 27, 2024. According to the weather forecast from AccuWeather, the outdoor temperature at the time the patient traveled from Hanoi to Phu Tho (Vietnam), around 40°C and 41°C, respectively, at 13:00–15:00; the humidity is unknown [5, 6].

He was discovered unconscious and taken to the nearest district hospital. The patient was not sweating on admission. He was admitted to the hospital for hyperthermia (41°C), coma (GCS: 3), and required oro‐tracheal intubation and mechanical ventilation. He was hypotensive and tachycardic despite 30 mL/kg of fluid, a normal saline bolus, high doses of norepinephrine (0.3) mcmol/kg/min, and a heart rate of 130/min. We had to give benzodiazepines to control seizures.

Arterial blood gas analysis revealed the following: pH (7.38), PCO_2_ (24 mmHg), PO_2_ (146 mmHg), HCO_3_^−^ (14.1 mmol/L), lactic acid (5.6 mmol/L), and anion gap (19 mmol/L) with FiO_2_ 40%, Peep 5 cm H20. A complete blood count revealed the following: White blood cell count (12.53 × 10^9^/L), hemoglobin (152.00 g/L), platelet count (153.00 × 10^9^/L), and neutrophil count (85%). The biochemical data were as follows: Blood urea nitrogen (15.4 mmol/L), creatinine (170.0 mcmol/L), creatine kinase (661 U/L), and interleukin 6 (1385 pg/mL), the alanine aminotransferase (ALT) (56 UI/L) and the aspartate aminotransferase (AST) (339 UI/L). The total bilirubin, direct bilirubin, and cardiac troponin I concentrations were within the normal ranges. The coagulation function test data were as follows: Prothrombin time (14.8 s), prothrombin activation (66%), international normalized ratio (1.34), activated partial thromboplastin time (14.8 s), and D‐dimer (5684 ng/mL), DIC Score: 5.

## 3 Methods

The targeted temperature management process can be divided into three phases: the induction phase, maintenance phase, and rewarming phase. The goal is to achieve a core temperature of 32°C–34°C as soon as possible, maintain this temperature for 12–24 h, and then rewarm at a controlled rate of 0.2–0.5 C/h based on the American Heart Association (AHA) recommends [7].

We rewarmed at a rate of 0.25°C/h to 37°C within 16 h, and the patient was taken off sedation to assess consciousness. After 2 days, the patient's consciousness improved from GCS 5 to 10, and the patient maintained a body temperature of 37°C for another 4 days.

**FIGURE 2:**
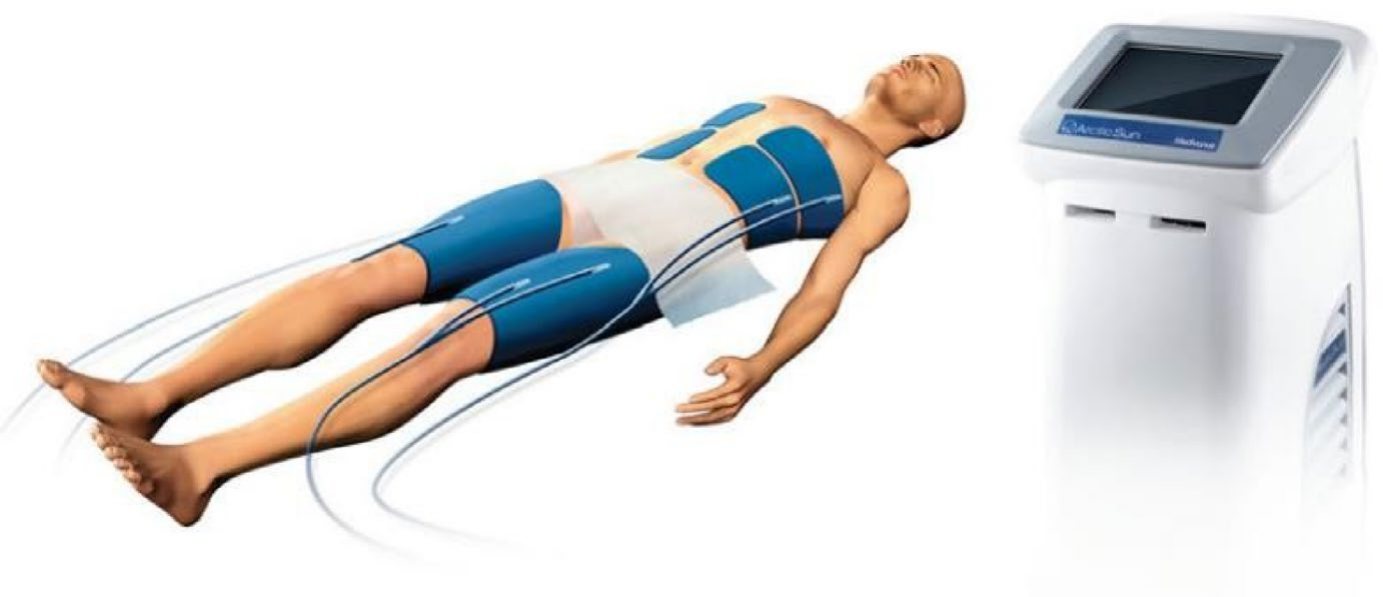
Arctic sun temperature management system (extravascular cooling devices (surface cooling) typically consist of several sheets or pads that wrap around the limbs or upper body and cool the skin directly. With circulating temperature‐controlled water within those pads, the system can regulate the patient's temperature. This is a non‐invasive method, and as a result, it minimizes the risk of bleeding or infection [8]).

**Chart 1:**
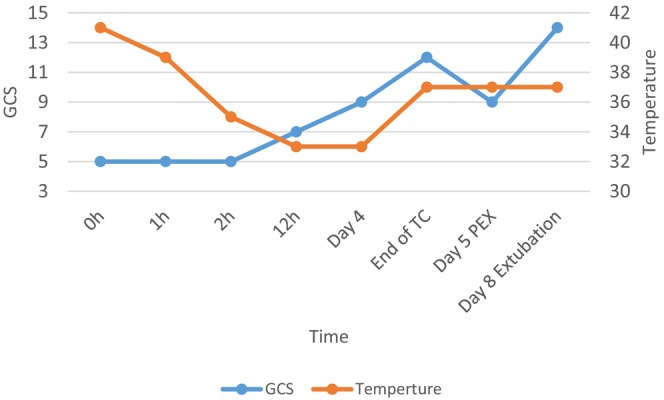
The patient's body temperature and Glasgow Coma Scale (GCS) after arriving at the emergency department (D0, Day of admission; D4, End of temperature control; D5, Before plasma exchange; D8, Extubation; on the 5th day after temperature control, the patient's consciousness decreased from GCS: 12 to GCS: 9; plasma exchange was performed). Abbreviations: PEX, Plasma exchange; TC, Temperature control.

When arriving at our department of intensive care & poisoning, the patient was treated with external cooling (ice packs, cold water spraying, and continuous fanning). Brain multi‐slice computed tomography (MSCT) was performed to exclude a cerebrovascular event. Meanwhile, the patient's body temperature dropped to 39.5°C, his level of consciousness remained at the GCS of 5. To accelerate cooling, an extravascular temperature control device (Figure 2) (Arctic Sun Temperature Management System, Medivance, Louisville, CO, USA) was applied. Extravascular temperature control was started 1 h after entering our department, and his body temperature dropped to 38.3°C 1 h after the external cooling (Chart 1). After 2 h, when the temperature dropped to 35°C, the patient showed signs of reacting to painful stimuli, but after 4 h, when the temperature dropped to 33°C, the patient still had no other response. After 12 h, the patient showed signs of irritation, resisted breathing, and responded to painful stimulation but still did not follow orders. We decided to maintain sedation to maintain hypothermia of 33°C within 24 h. The patient was then warmed at a rate of 0.25°C/h to 37°C, taking 16 h, and sedation was stopped.

**Chart 2:**
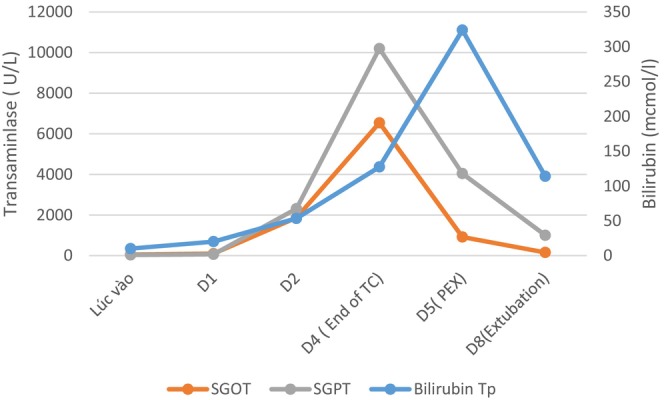
Graph shows liver function at different points of time. (D0, Day of admission; D4, End of temperature control; D5, Before plasma exchange; D8, Extubation).

After 24 h, the patient was in a coma, GCS: 10, no weakness, and the patient maintained a temperature of 37°C for the next 4 days. Several attempts to stop the active cooling within this period (Figure 1) led to an immediate steep increase in core body temperature, which forced us to prolong this very efficacious treatment. On day 5, liver failure progressed, consciousness decreased, and the patient received plasma exchange (Chart 2) and coagulation disorder (Table 1). The patient was extubated on day 8 and discharged without reports of neurological complications on day 24.

**TABLE 1:** The patient's coagulation and kidney function.

	D0	D1	D2	D4	D5	D8
INR	1.98	1.83	2.53	1.56	3.96	1.35
APTT (s)	36.3	38.4	45	44.9	52	41
Fibrinogen (g/L)	3.5	1.81	1.58	1.35		2.89
D‐Dimer	5600	7996	15,580	1350		
Platelet (G/L)	153	80	46	88	72	181
DIC Score	5	6	7			
Ure (mmol/L)	6.1	5	5.2	5.5	4.7	4
Cre (mcmol/L)	170	121	100	96	72	67

*Note:* At admission time, the lab test showed he had kidney failure and the index was improved by time.

Abbreviations: APTT, activated partial thromboplastin time; Cre, Creatinine; D0, Day of admission; D4, End of temperature control; D5, Before plasma exchange; D8, Extubation; DIC, Disseminated intravascular coagulation; INR, International normalized ratio.

## 4 Conclusion

This clinical case demonstrates the effective use of extravascular temperature control in treating heatstroke, particularly in a young adult patient. The patient's body temperature was successfully reduced from 41°C to 33°C within 24 h, followed by a controlled rewarming process to 37°C over 16 h. This intervention contributed to the improvement of the patient's consciousness and stabilization of body temperature. Despite initial complications such as liver failure and severe coagulation disorders, targeted treatments including fresh frozen plasma and plasma exchange facilitated recovery. The patient was eventually extubated after 8 days and discharged after 24 days of comprehensive care. This case underscores the importance of combining temperature management with supportive treatments for organ damage in the effective management of heatstroke.

The patient was re‐examined after 1 month, and laboratory test results returned to normal values: Blood count (7.5 × 10^9^/L), hemoglobin (150 g/L), platelet count (371 × 10^9^/L), urea (4.3 mmol/L), creatinine (72.9 mcmol/L), creatine kinase (74 U/L), ALT (22 UI/L), AST (26 UI/L), total bilirubin (13 mcmol/L), direct bilirubin (5.9 mcmol/L). Coagulation profile: prothrombin time (11 s), prothrombin activity (100%), international normalized ratio (1.0), activated partial thromboplastin time (30.2 s), and D‐dimer (532 ng/mL). The patient was scheduled for a follow‐up examination in 3 months.

## 5 Discussion

Even when the environmental temperature changes, the temperature of human body should be maintained constantly through the hypothalamic thermoregulation function. However, continuous high temperatures from outside, excessive physical activity in hot environments, or direct sunlight damage to the thermoregulatory center can lead to body thermoregulation disorders. Heatstroke is a potentially life‐threatening illness for which there is no effective pharmacological treatment. Death can occur due to multi‐organ dysfunction, such as cerebral edema and liver and kidney failure. A poorer prognosis is generally associated with long‐term exposure to slightly lower temperatures than with short‐term exposure to high temperatures. In this case, the patient rode on the motorbike in the sun at high temperatures for around 2 h, causing disturbances in the thermoregulation center and/or damage to the thermoregulation center, causing heatstroke.

In terms of heatstroke management, in 2023, the American Heart Association (AHA) recommends [9] that with heatstroke treatment, during post‐arrest temperature control, a constant temperature between 32°C and 37.5°C should be maintained (Class 1). It is reasonable that temperature control should be maintained for at least 24 h after achieving target temperature (Class 2a), and patients with spontaneous hypothermia after ROSC unresponsive to verbal commands should not routinely be actively or passively rewarmed faster than 0.5°C per hour (Class 2b). The routine use of rapid infusion of cold intravenous fluids for prehospital cooling of patients after ROSC is not recommended.

Targeted temperature management (TTM) use has been demonstrated in several trials to enhance neurological function and survival [10, 11]. Surface cooling systems, intravascular cooling systems, and conventional cooling techniques are the three primary types of cooling procedures [7]. *Conventional cooling techniques*: The simplest and most economical techniques include the use of ice packs and cold saline infusions. The drawbacks of conventional technologies are their high labor costs, tendency to produce temperatures below the desired level, and inefficiency in sustaining the target temperature [12]. *Surface cooling systems*: Surface cooling systems circulate cold air or fluid by wrapping the patient in blankets or pads. The uncommon danger of skin burns and irritation (redness and mottling) as well as the possibility of exceeding the target temperature during the induction phase are disadvantages. *Core cooling systems*: These mostly comprise of intravascular catheters that circulate cold saline and are inserted into a major vein. The intrusive nature of intravascular cooling devices, the risk of catheter‐related infection and thrombosis, and their comparatively high cost are their disadvantages.

Temperature management can also classify to external cooling methods (e.g., immersion in water) and internal cooling methods (e.g., gastric lavage, bladder, and rectum with cold water or cold isotonic saline intravenously). These treatments should be stopped when the body temperature reaches approximately 39°C because it is difficult to accurately determine the body temperature using these methods [2]. Intravascular or extravascular temperature control may be used, which is commonly used in the treatment of coma after cardiac arrest [4]. There are very few reports of the use of this device to treat heatstroke. In the two cases reported by Hong et al. [13] and Lee et al. [14], using an external cooling device to successfully treat heatstroke, the body temperatures of the treated patients were lowered to 33°C. The report by author Byung‐Chan Lee [15], lowering the patient's temperature to a target of 36.6°C due to the patient's rapid improvement in consciousness during the hypothermia process, the patient opened his eyes naturally when the temperature dropped 36.5°C, so the authors maintained it for an additional 12 h before ending hypothermia. Three reports showed positive results, and she was discharged without any neurological complications. In our case, the patient was brought down to a target temperature of 33°C, as during hypothermia, the patient's consciousness progressed slowly and the GCS increased by 3–6 points, so we decided to maintain the target temperature of 33°C within 24 h before rewarming and stopping sedation.

Besides, a definition of heatstroke was presented, implying that the combined effects of heat cytotoxicity, coagulopathies, and a systemic inflammatory response syndrome were responsible for multi‐organ system failure [2]. An increase in epidermal blood flow that encourages heat escape and lowers the rate of heat uptake from the surroundings is the main cardiovascular reaction to heat exposure. As a compensatory strategy to maintain blood pressure, increased cutaneous blood flow is accompanied by decreased splanchnic blood flow. Reduced cerebral blood flow (CBF) is another effect of hyperthermia that might explain presyncopal symptoms or anomalies in the central nervous system [16]. The SIRS is thought to be a reaction to bacterial infection that develops when long‐term decreases in splanchnic blood flow cause harm to the stomach and other organs. The resulting ischemia environment encourages oxidative and nitrosative stress, which makes the gut's tight junctions “leaky.” The tight connection barrier, therefore, permits both Gram‐positive and Gram‐negative bacteria, which are typically found in the gut lumen, to readily pass through and enter the systemic circulation [17]. Since the liver is a key organ for endotoxin clearance, there may be a link between endotoxin levels in the blood and liver damage in both heatstroke patients and animal models [18].

Our patient had multi‐organ damage at the time of admission but recovered rapidly thereafter. Some studies reported a higher degree of organ damage expressed as the diabetic foot [19], a vascular impairment [20, 21]. In addition, immune‐inflammatory marker and tumor necrosis factor (TNF)‐α, interleukin (IL‐6), and IL‐1β plasma levels are significantly related to ischemic lesions [22]. The patient had acute liver failure, which is characterized by liver injury (abnormal liver tests), coagulopathy (international normalized ratio [INR] > 1.5), and hepatic encephalopathy occurring on the 5th day after admission. The patient was initially treated with a plasma exchange procedure [23]. One day of plasma exchange showed significantly improving AST, ALT, and total bilirubin levels and the international normalized ratio [INR], as well as the patient's neurological status.

Heat‐related illnesses and fatalities can be avoided [2]. Optimal treatment in heatstroke patients relies on early recognition and expedition of rapid cooling [24]. Aging, previous infection, body composition, sex differences, and dehydration are potential risk factors for exertional heatstroke. Interestingly, exertional heatstroke is more prevalent in young cohorts [25]. Wearing loose‐fitting, light‐colored clothing, limiting outdoor activities during the day, using air‐conditioning, drinking lots of water, being aware of medication side effects that can cause dehydration, decreased sweating, or a low heart rate, and never leaving an adult or child unattended in a car are all important ways to prevent heatstroke. La Isla Network, which was founded with the mission to help workers exposing to risks in the workplace driven by climate change, issued HASTE that is ways to detect and promptly act with heatstroke. HASTE includes H—Heat exposure identify; A—Altered mental status; S—Start Cooling the patient immediately; T—Time, recognize the urgency; E—Emergency actions to prevent potential complications and ensure the best possible outcome [26]. There are plenty of efforts to improve people's health literacy about heatstroke and reduce the time of detection and treatment, which should be continued and support, especially in such hot weather nations as Vietnam.

Intravenous fluids, vital sign monitoring, and boosting heat loss (by taking off clothes and other cooling techniques) are examples of immediate treatments. Given that heat exhaustion can occasionally develop into heatstroke, it is crucial to monitor body temperature and loss of consciousness [27].

In conclusion, heatstroke is a severe condition that affects multiple organs and necessitates timely intervention. The treatment of heatstroke involves a combination of organ function support and cooling strategies. Extravascular body temperature control has emerged as a promising approach for managing the body temperature of individuals suffering from heatstroke. This method is perceived to be more convenient and efficient compared to traditional cooling techniques like immersion in cold water, cold saline infusion, and gastric or bladder lavage with cold water. By utilizing extravascular body temperature control, it is possible to effectively regulate and maintain the body temperature within the desired range. Nonetheless, further research is essential to fully evaluate the efficacy and potential benefits of this method in heatstroke management.

## Author Contributions

**Van Ha Thi Bich:** conceptualization, data curation, formal analysis, methodology, project administration, resources, supervision, validation. **Lich Nguyen Duc:** formal analysis, investigation, resources, software, visualization, writing – original draft, writing – review and editing. **Anh Tran Ngoc:** data curation, formal analysis, investigation, writing – original draft, writing – review and editing.

## Ethics Statement

Hereby, I, Van Ha Thi Bich consciously assure that the manuscript is the authors' own original work, which has not been previously published elsewhere. The paper reflects the authors' own research and analysis in a truthful and complete manner. All subjects gave their informed consent for inclusion before they participated in the study. Our research was conducted following ethical guidelines, and all necessary approvals were obtained from the Boards of Directors of Phu Tho Provincial General Hospital.

## Consent

Written informed consent was obtained from the patient to publish this report in accordance with the journal's patient consent policy.

## Conflicts of Interest

The authors declare no conflicts of interest.

## Data Availability

The data that support the findings of this study are available from the corresponding author upon reasonable request.
